# Tissue transglutaminase in astrocytes is enhanced by inflammatory mediators and is involved in the formation of fibronectin fibril-like structures

**DOI:** 10.1186/s12974-017-1031-2

**Published:** 2017-12-28

**Authors:** Nathaly Espitia Pinzón, John J. P. Brevé, John G. J. M. Bol, Benjamin Drukarch, Wia Baron, Anne-Marie van Dam

**Affiliations:** 1 Department Anatomy and Neurosciences, VU University Medical Center, Amsterdam Neuroscience, De Boelelaan 1108, 1081 HZ Amsterdam, The Netherlands; 2 Department of Cell Biology, University Medical Center Groningen, Groningen, The Netherlands

**Keywords:** Tissue transglutaminase, Neuroinflammation, Astrocytes, Astroglial scarring, Extracellular matrix, Fibronectin

## Abstract

**Background:**

During multiple sclerosis (MS) lesion formation, inflammatory mediators are produced by microglial cells and invading leukocytes. Subsequently, hypertrophic astrocytes fill the lesion and produce extracellular matrix (ECM) proteins that together form the astroglial scar. This is beneficial because it seals off the site of central nervous system (CNS) damage. However, astroglial scarring also forms an obstacle that inhibits remyelination of brain lesions. This is possibly an important cause for incomplete remyelination of the CNS in early stage MS patients and for failure of remyelination when the disease progresses. Tissue transglutaminase (TG2), a Ca^2+^-dependent enzyme that can cross-link proteins, appears in astrocytes in inflammatory MS lesions and may contribute to the rearrangement of ECM protein deposition and aggregation.

**Methods:**

The effect of different inflammatory mediators on TG2 and fibronectin, an ECM protein, protein levels was examined in primary rat microglia and astrocytes by western blotting. Also, TG2 activity was analyzed in primary rat astrocytes by a TG activity assay. To determine the role of TG2 in the deposition and cross-linking of fibronectin, a TG2 inhibitor and TG2 knockdown astrocytes were used.

**Results:**

Our data show that under inflammatory conditions in vitro, TG2 production is enhanced in astrocytes and microglia. We observed that in particular, astrocytes produce fibronectin that can be cross-linked and aggregated by exogenous TG2. Moreover, inflammatory stimulus-induced endogenously produced TG2 is involved in the appearance of morphological fibril-like fibronectin deposits but does not lead to cross-linked fibronectin aggregates.

**Conclusions:**

Our in vitro observations suggest that during MS lesion formation, when inflammatory mediators are produced, astrocyte-derived TG2 may contribute to ECM rearrangement, and subsequent astroglial scarring.

**Electronic supplementary material:**

The online version of this article (10.1186/s12974-017-1031-2) contains supplementary material, which is available to authorized users.

## Background

In multiple sclerosis (MS), a chronic inflammatory, demyelinating disease of the central nervous system (CNS), various classes of inflammatory white matter lesions can be identified [1, 2]. Active white matter lesions appear with a massive influx of leukocytes and ongoing demyelination. In these lesions, e.g., inflammatory cytokines and chemokines are produced by activated microglial cells and invading leukocytes [1, 3–6]. In chronic MS lesions, infiltrating cells, particularly macrophages, remain present at the rim of the lesion where demyelination is still ongoing [7]. Simultaneously, astrogliosis appears in the lesion which mainly consists of astrocytes with a hypertrophic phenotype [8–11]. The formed astroglial scar is beneficial because it seals of inflammation in the CNS into focal areas [12]. However, the scar also impedes remyelination by inhibiting the migration and differentiation of oligodendrocyte precursor cells [13]. Remyelination in MS lesions is therefore not very effective [14–16], which might contribute to the chronic neurodegenerative character of this disease with progressive loss of motor, sensory, and cognitive functions [17].

During astrogliosis, the extracellular matrix (ECM) is largely modified, which is reflected by an enhanced production of ECM proteins by astrocytes [18–22]. Previous studies, under in vitro conditions, in MS animal models and in brain material from patients already showed changes in the production and deposition of chondroitin sulfate proteoglycans, a family of ECM proteoglycans [23, 24]. Furthermore, the ECM proteins fibronectin and laminin have been shown to be more expressed by astrocytes [25–28] and aggregated in chronically demyelinated multiple sclerosis lesions [29], possibly contributing to the non-regenerative nature of these lesions [30, 31].

Tissue transglutaminase (TG2) is a Ca^2+^-dependent enzyme with various catalytic functions, including protein deamidation, transamidation, and cross-linking [32–38]**.** TG2 is localized intracellularly [39], on the cell surface and in the ECM [40, 41]. In the ECM, TG2 is capable of cross-linking a wide range of proteins, which are important in ECM deposition and stabilization [42, 43]. TG2 can also directly, i.e., non-enzymatically, interact with fibronectin, an important ECM protein in tissue repair processes, and various beta-integrins, thereby mediating cell-ECM interactions [44–46]. TG2 is therefore thought to play an important role in various physiological and pathological situations including inflammation and fibrosis [36, 47] and can possibly contribute to the process of astroglial scarring in the CNS of MS patients and other brain injuries.

We have previously shown the appearance of TG2 immunoreactivity in astrocytes in active and chronic active MS lesions [48] and in demyelinating areas in the mouse brain after cuprizone treatment [49]. Moreover, TG2 expression in astrocytes is regulated by some inflammatory mediators as are present in MS lesions [50]. In the present study, we questioned whether a wide array of inflammatory mediators can affect TG2 production in astrocytes and possibly also in microglia which can be a source of ECM proteins during the formation of the astroglial scar [51–56]. We also questioned whether TG2 contributes to the production, deposition, and cross-linking of the ECM protein fibronectin under inflammatory conditions. A better understanding of the inflammatory regulation of TG2 and its role in the production, deposition, and cross-linking of ECM proteins by astrocytes and possibly microglia could be of therapeutic interest to overcome astroglial scarring and promote a remyelinating milieu.

## Methods

### Primary astrocytes and microglia

Primary rat glial cells were isolated from cerebral cortices of 2-day-old Wistar rats (Harlan CPB, Zeist, The Netherlands), as described previously [57], and approved by the Animal Experiment Committee of the VU University Medical Center (ID FGA 11-03). The meninges and blood vessels were removed from the cortices. Cortices were mechanically homogenized in Dulbecco’s Modified Eagle’s Medium-F10 (Gibco, Life Technologies, Breda, The Netherlands), supplemented with 10% *v*/*v* heat-inactivated fetal calf serum (Gibco), 2 mM L-glutamine (Sigma-Aldrich, St. Louis, MO, USA), 50 U/ml penicillin (Sigma-Aldrich), and 50 μg/ml streptomycin (Gibco) to make a cell suspension. Dissociated cells from 2 to 3 pups were plated in poly-L-lysine (PLL, 15 μg/ml (2 μg/cm^2^); Sigma-Aldrich) coated T75 culture flasks (Nunc, Hamstrop, Denmark) and incubated at 37 °C in humidified air containing 5% CO_2_. The medium was changed at days 1, 6, and 8 after seeding. After 10 days in culture, microglia and astrocytes were separated by shaking the flasks on a rotary platform (Heidolph Unimax 2010) at 230 rpm for 16 h. Astrocytes were further purified by treatment with 5 mM leucine methyl ester (Sigma-Aldrich) in serum-free medium, for 24 h at 37 °C. Microglia were cultured in fresh medium mixed with conditioned medium (1:1 ratio) collected from the mixed cell culture before separation. The purity of astrocyte cultures was between 80 and 90%, as described previously [58], determined by immunofluorescent staining using glial fibrillary acidic protein (GFAP) antibody (1:6000; DAKO, Glostrup, Denmark). Purity of primary microglial cultures was between 90 and 94% as determined by immunofluorescent staining using Iba1 antibody (1:1000; WAKO Chemicals USA).

### Treatments

Astrocytes and microglia cultured on 2 μg/cm^2^ PLL-coated 6-well plates (Nunc) were incubated for 48 h in medium alone (control) or with lipopolysaccharide (LPS, *E. coli* 055-B5, Difco, 100 ng/ml), human recombinant (rec) transforming growth factor (TGF)-β1 (BioLegend), human rec TGF-β2 (BioLegend), rat rec interleukin (IL)-1β (Glaxo), rat rec tumor necrosis factor (TNF)-α (Biolegend), rat rec TNF-α+IL-1β, rat rec interleukin (IL)-4 (BioVision), rat rec interleukin (IL)-6 (gift from Steve Poole), or rat rec interleukin (IL)-10 (Pharmingen) (all 20 ng/ml). In addition, astrocytes and ECM deposited by astrocytes were treated for 48 h with TNF+IL-1β in the absence or presence of 10 μM of the bona fide dihydroisoxazole TG2 activity inhibitor ERW1041E (Quinolin-3-ylmethyl(S)-2-((((S)-3-bromo-4,5-dihydro-isoxazol-5-yl)methyl)carbamoyl)pyrrolidine-1-carboxylate) diluted in 0.1% (*v*/*v*) dimethyl sulfoxide (DMSO); kind gift from C. Khoshla, Stanford University, USA) [59–61]. Astrocytes were alternatively treated with exogenous recombinant guinea pig TG2 (Sigma-Aldrich) in pathophysiological concentrations (0.13 and 1.3 μM) according to previously published studies [62, 63] for 48 h. Additionally, deposited ECM, after removal of astrocytes, was treated with 0.64 μM exogenous recombinant guinea pig TG2 (Sigma-Aldrich) for 16 h combined with a pre-incubation with a selective inhibitor of TG2 (Z-DON, 1 μM, Zedira) diluted in 0.001% DMSO or only with DMSO for 30 min (min) at 37 °C. In some experiments, astrocytes were cultured in 8-well Lab-Tek Permanox chamber slides (Nunc) or 6-well plates (Nunc) coated with 2 μg/cm^2^ laminin (mixture of laminin-1 and laminin-2, from Sigma-Aldrich) instead of PLL.

### Lentiviral downregulation of TG2

TG2 was downregulated in primary rat astrocytes by lentiviral transduction with TG2 specific shRNA. Therefore, astrocytes were plated on a laminin (2 μg/cm^2^; Sigma-Aldrich) coated 12-well plate (0.2 × 106 cells/well, Nunc). The following day, 0.2 × 106 infectious units of virus (IFU) of lentiviral particles (Santa Cruz, sc-270266-V for rat TG2 shRNA, or sc-108080 for control scrambled shRNA) were added to the cells. The next day, the same amount of lentiviral particles was added to the cells. The medium was changed the following day, and the cells were left for 5 days before treatment with TNF-α+IL-1β.

### Sample collection

#### Whole cell lysates

Whole cell lysates were obtained by homogenizing the cells in ice-cold lysis buffer containing 20 mM tris-HCl pH 7.5, 137 mM NaCl, 1 mM ethylenediaminetetraacetic acid, 1% NP40, 1% sodium deoxycholate, 0.1% sodium dodecyl sulfate (SDS), 50 mM dithiothreitol (DTT), 100 μM phenylmethanesulfonyl fluoride (PMSF), 7.5 μM pepstatin-A, 10 μM leupeptin, and 0.75 μM aprotinin (all from Sigma-Aldrich).

#### Transglutaminase activity lysates

For transglutaminase (TG) activity measurements, cells were collected in tris-buffered saline (TBS), pH 7.5, containing 100 μM PMSF, 7.5 μM pepstatin-A, 10 μM leupeptin, and 0.75 μM aprotinin (all from Sigma-Aldrich).

#### ECM lysates

For the collection of ECM samples, cells were removed after washing with TBS at room temperature (RT), by incubation (5 min) with 50 mM NH_4_OH + 0.05% Triton-100 (Sigma-Aldrich). This was followed by incubation (5 min) with 50 mM NH_4_OH. Any remaining cell fractions were washed away by repeated wash steps with TBS. The ECM was incubated for 1 h at 37 °C with DNAse (10 U/ml, Promega) in a buffer (pH 7.9) containing 4.84 g/L tris, 0.58 g/L NaCl, 0.57 g/L MgCl_2_, and 1.95 g/L CaCl_2_. The ECM was again washed with TBS and collected in ice-cold lysis buffer as described for the whole cell lysates.

### Sample preparation

After sample collection, homogenates of whole cell lysate samples and TG activity samples were sonicated (Branson “Sonifier 250”; output 1, duty 30%, 8 pulses) and cleared by centrifugation (20,000 g for 10 min at 4 °C), and protein concentrations of supernatants were determined by the BCA method (Pierce Biotechnology, Perbio Science, Etten-Leur, The Netherlands). Samples were heated for 10 min at 95 °C. ECM samples were not subjected to sonication or heating to maintain the integrity of the ECM aggregates.

### Western blotting

Equal amounts of protein (20 μg) were subjected to 8% SDS-polyacrylamide gel electrophoresis and transferred to a nitrocellulose membrane (Li-Cor Biosciences, Lincoln, NE, USA). Membranes were blocked at RT with Odyssey blocking buffer (Li-Cor Biosciences) diluted 1:1 with TBS. Membranes were incubated overnight at 4 °C with mouse anti-TG2 (ab3, Neomarkers 1:2000), rabbit anti-fibronectin (Neomarkers, 1:1000), or mouse anti-β-actin (Abcam, 1:5,0000) antibodies. After several washes with TBS containing 0.1% triton (TBS-T), blots were incubated for 1 h at RT with corresponding goat anti-rabbit or donkey anti-mouse IRDye 800CW IgG’s (1:10,000, Li-Cor Biosciences) for subsequent antigen detection. All antibody solutions were prepared in the Odyssey blocking buffer (Li-Cor Biosciences) diluted 1:1 with TBS-T. After washing with TBS-T and TBS, membranes were scanned for fluorescence emission at 800 nm, using an Odyssey infrared imaging system (Li-Cor Biosciences). Bands were visualized, and their signal intensities were measured using the Odyssey Sa Infrared scanning software (version 1.1, Li-Cor Biosciences).

### Transglutaminase activity assay

TG activity was measured using the commercially available TG Covtest Transglutaminase Colorimetric Microassay (Covalab, Villeurbanne, France), which uses immobilized CBZ-Gln-Gly as the first substrate and biotinylated-cadaverine (biotin-Cd) as second substrate of the enzyme. To determine the amount of TG2 activity among the TG activity measured, samples were pre-incubated with a selective inhibitor of TG2 (Z-DON, 1 μM, Zedira) diluted in 0.001% DMSO or only with DMSO for 30 min at RT. The assay was performed following the manufacturer’s instructions [64]. In brief, 10 μg/well of each sample was incubated with 50 μl/well of biotin-Cd solution containing CaCl_2_ for 1 h at 37°C on the CBZ-Gln-Gly coated plates. At the end of the incubation period, plates were washed three times with TBS (pH 7.5) containing 0.1% Tween 20. Then, 100 μl/well of streptavidin-labeled horseradish peroxidase (HRP) diluted to 1:2000 was added to the wells and incubated for 1 h at RT. After washing, peroxidase activity was revealed using 100 μl/well of 0.01% H_2_O_2_ as HRP substrate and (0.1 mg/ml) tetramethylbenzidine as electron acceptor (chromogen). The reaction was stopped by the addition of 2.5 N H_2_SO_4_, and TG activity was detected by absorbance measurement of streptavidin-labeled peroxidase activity in each well on a microplate reader (SpectraMax 250, Molecular Devices, Sunnyvale, CA, USA) at 450 nm. Guinea pig TG2 (T5398, Sigma-Aldrich) was used as a standard. One unit of guinea pig TG2 will catalyze the formation of 1.0 μmole of hydroxamate per min from Na-Z-Gln-Gly and hydroxylamine at pH 6.0 at 37 °C.

### Immunocytochemistry

Astrocytes (4.0 × 104/well) were plated on laminin (2 μg/cm^2^, Sigma-Aldrich) or PLL (2 μg/cm^2^, Sigma-Aldrich) coated 8-well Lab-Tek Permanox chamber slides (Nunc) and treated with TGF-β1 (for ECM stainings only) or a combination of TNF-α + IL-1β cytokines (20 ng/ml each) for 48 h. Alternatively, exogenous recombinant guinea pig TG2 (0.13 and 1.3 μM, Sigma-Aldrich) was added for 48 h to untreated astrocytes. Astrocytes were fixed in ice-cold methanol for 10 mins and washed in TBS. Nonspecific binding was blocked with 3% bovine serum albumin in TBS containing 0.05% triton (TBS-T, pH 7.6) for 30 min at RT. Subsequently, astrocytes were double-labeled with mouse anti-TG2 (ab1, Neomarkers, 1:300) and the astrocytic marker rabbit anti-GFAP (DAKO, 1:4000) or with rabbit anti-fibronectin (Millipore, 1:200) to examine TG2 localization in astrocytes and any co-localization with fibronectin, respectively. Triton was alternatively omitted from all the incubation steps during double stainings to detect TG2 and fibronectin on the cell surface. Prior to ECM stainings, astrocytes were removed as described before. Triton was not omitted for the staining of the ECM. Secondary antibodies used were the appropriate Alexa Fluor© (488 or 594)-conjugated IgG’s (Molecular Probes, 1:400). After washing in TBS, astrocytes were cover-slipped in polyvinyl alcohol mounting medium with DABCO (Sigma-Aldrich) and examined using a confocal microscope (Leica TSC-SP2-AOBS; Leica Microsystems, Wetzlar, Germany).

### Filter trap assay

ECM lysates were treated for 30 min at RT with 150 mM DTT to break down disulfide bonds and to obtain stable aggregates. Equal amounts of ECM samples (12 μg) were applied to a cellulose acetate membrane (GE water and process technologies) using a filter trap assay apparatus (FTA, Bio-Dot SF microfiltration apparatus, Bio-Rad). This apparatus was coupled to a vacuum-pump in order to wash through the monomers and dimers of ECM aggregates and trap aggregates larger than the pore-size of 5 μm of the membrane. Approximately 5 min after sample application, the membrane was washed in PBS and incubated overnight at 4 °C with rabbit anti-fibronectin (Neomarkers, 1:1000) antibodies. For subsequent antigen detection, blots were incubated for 2 h with corresponding goat anti-rabbit IRDye 800CW IgG’s (1:10,000, Li-Cor Biosciences). After washing with TBS-T and TBS, membranes were scanned for fluorescence emission at 800 nm, using the same Odyssey infrared imaging system (Li-Cor Biosciences) as used for western blotting.

### Statistical analysis

Normal distribution of the data was tested using the Shapiro-Wilk procedure. One-way analysis of variance was performed followed by Dunnett’s post hoc test for multiple comparisons by using IBM SPSS software, version 20.0 (IBM Corp., NY, USA). *P* < 0.05 was considered to represent statistically significant differences, and a *P* value between 0.05 and 0.1 was regarded as a statistical trend.

## Results

### Astrocytes had higher TG2 and fibronectin protein levels than microglia

**Fig. 1:**
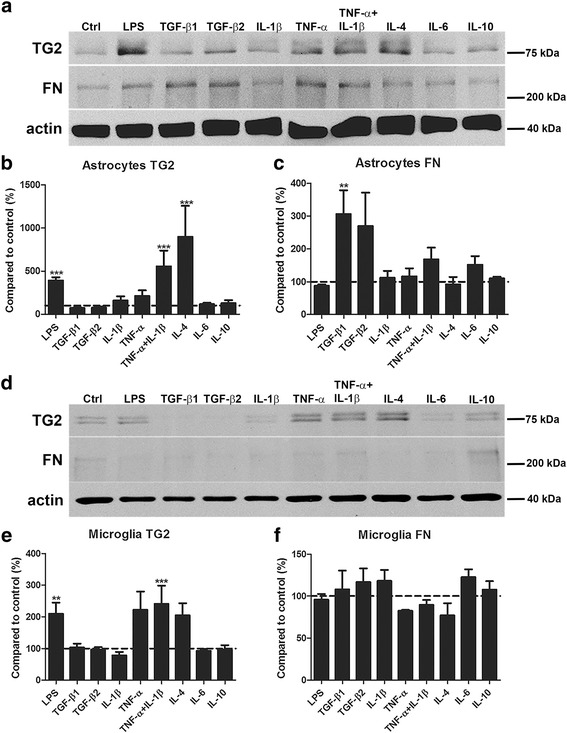
TG2 and fibronectin protein levels in astrocytes and microglia. Primary rat astrocytes and microglia were treated for 48 h with a range of inflammatory mediators. TG2 and fibronectin (FN) protein levels in astrocytes were detected by western blotting (**a**), which were subsequently semi-quantified (**b, c**). TG2 and fibronectin (FN) protein levels in microglia were detected by western blotting (**d**), which were subsequently semi-quantified (**e, f**). Representative blots of three independent experiments are shown. Each bar represents the mean + standard error of the mean of signal intensities from blots of three separate experiments that were calculated as average percentage compared to control (**p < 0.01 and *** *p* < 0.001)

We first examined whether primary rat astrocytes and microglia produced TG2 and fibronectin. Under control conditions and after treatment with LPS, astrocytes expressed more TG2 and fibronectin than microglia (Additional file 1). Upon subsequent treatment of astrocytes and microglia with a variety of inflammatory mediators, TG2 protein levels were significantly increased after LPS, TNF-α+IL-1β, or IL-4 treatment (*p* < 0.001 for all, Fig. 1a, b). Little protein was found in untreated microglia. Incubation of microglia with the various mediators resulted in significant changes in TG2 protein levels (Fig. 1d, e). LPS or TNF-α+IL-1β treatment resulted in a significant increase in TG2 protein level (*p* < 0.01 for LPS and *p* = 0.001 for TNF-α+IL-1β, Fig. 1d, e). Moreover, IL-4 treatment showed a trend (*p* = 0.080) toward an increase in TG2 protein level (Fig. 1e). Other conditions studied, i.e., TGF-β1, TGF-β2, IL-1β, TNF-α, IL-6, and IL-10, did not show significant effects on TG2 protein level in astrocytes or microglia (Fig. 1a–e). Double bands at approximately 78 kDa were visible and quantified for TG2 in most conditions studied. This has been shown by others as well [65, 66] and could represent the full-length TG2 protein and the short TG2 protein that is truncated at the 3′ end [67]. Fibronectin levels were significantly increased in astrocytes after treatment with TGF-β1 (*P* < 0.01, Fig. 1a, c), and TGF-β2 showed a trend (*P* = 0.062, Fig. 1c) toward an increased fibronectin level. Other conditions studied, i.e., LPS, IL-1β, TNF-α, TNF-α+IL-1β, IL-4, IL-6, and IL-10, did not result in significant effects on fibronectin protein levels in astrocytes (Fig. 1a, c). Moreover, none of the treatments affected the level of fibronectin in microglia (Fig. 1d, f). Considering the minimal amount of fibronectin detected in microglia (Fig. 1d), we decided to continue our study with a focus on astrocytes.

### TG2 expression and activity in astrocytes were increased by inflammatory conditions

**Fig. 2:**
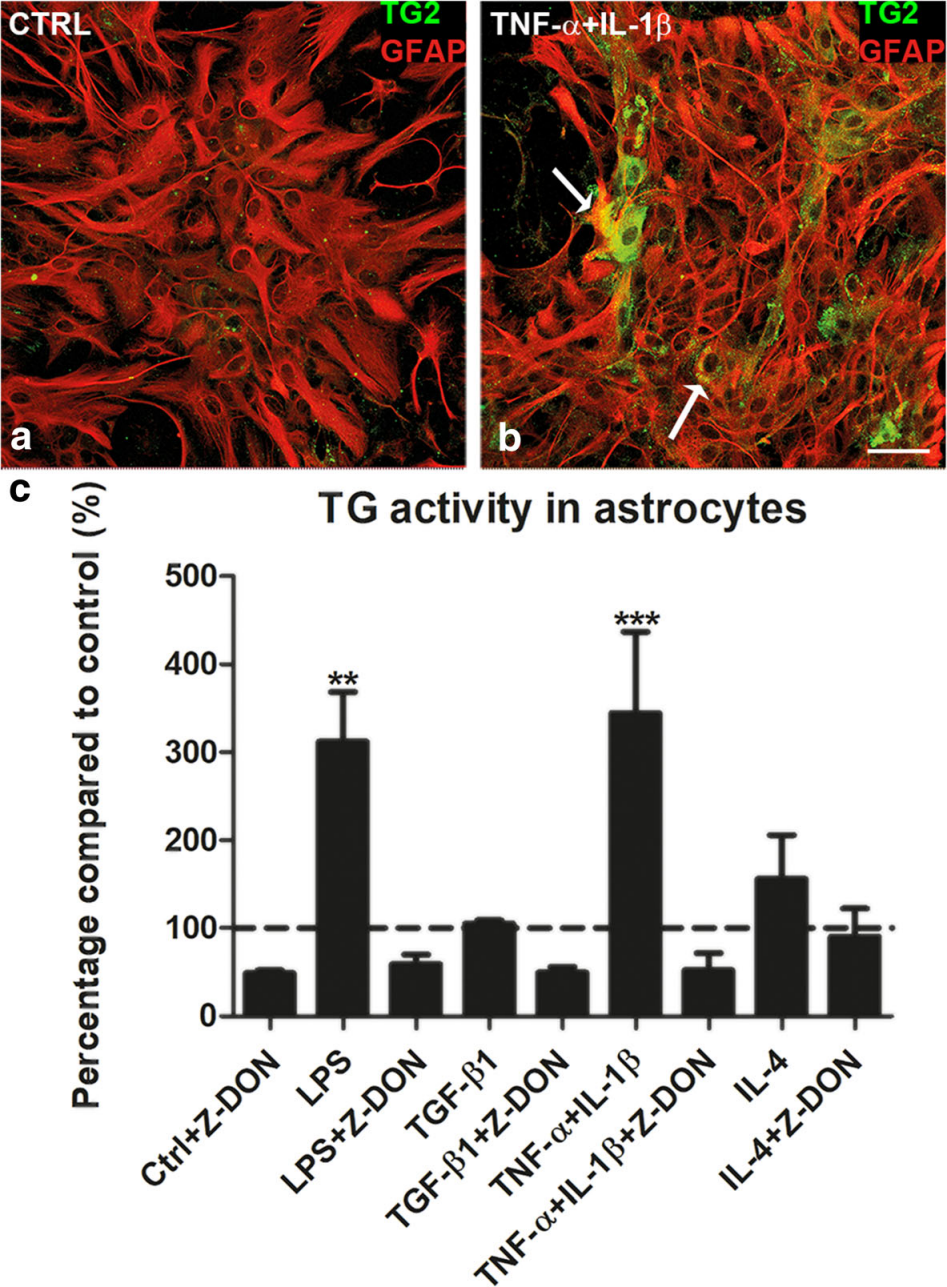
TG2 expression and activity in astrocytes. Primary rat astrocytes were treated for 48 h with medium alone as a control condition (CTRL) (**a**) or with TNF-α+IL-1β (**b**). TG2 immunoreactivity (green) was detected in astrocytes (as indicated by arrows in **b**) which were visualized by the astrocytic marker glial fibrillary acidic protein (GFAP, red). Representative images of three independent experiments are shown. Scale bar represents 50 μm. TG activity was examined in protein samples of astrocytes that were treated for 48 h with a range of inflammatory mediators. Subsequent derived astrocyte protein lysates were pre-incubated with a selective inhibitor of TG2 (Z-DON, 1 μM) diluted in 0.001% DMSO or only with DMSO for 30 min at room temperature (**c**). Each bar represents the mean + standard error of the mean of measurements from three separate experiments that were calculated as average percentage compared to control (**P < 0.01 and ***P < 0.001)

We subsequently visualized TG2 by immunohistochemistry in astrocytes. Under control conditions, little TG2 immunoreactivity was observed (Fig. 2a), but a clear increase was apparent in TG2 immunoreactivity in specific GFAP positive astrocytes after 48 h of TNF-α+IL-1β treatment (indicated by arrows in Fig. 2b). Inflammatory conditions not only affected expression of TG2 in astrocytes, but also increased its activity. In particular, LPS (*P* < 0.01) and TNF-α+IL-1β (*P* < 0.001) increased TG activity by astrocytes (Fig. 2c) which was reduced when adding the TG2 specific inhibitor Z-DON to the activity assay, indicating that the measured TG activity is mainly due to TG2. Of interest is that although IL-4 increased TG2 protein level, it showed a trend but did not significantly affect TG activity (Fig. 2c).

### TG2 and fibronectin were deposited in the ECM by astrocytes

**Fig. 3:**
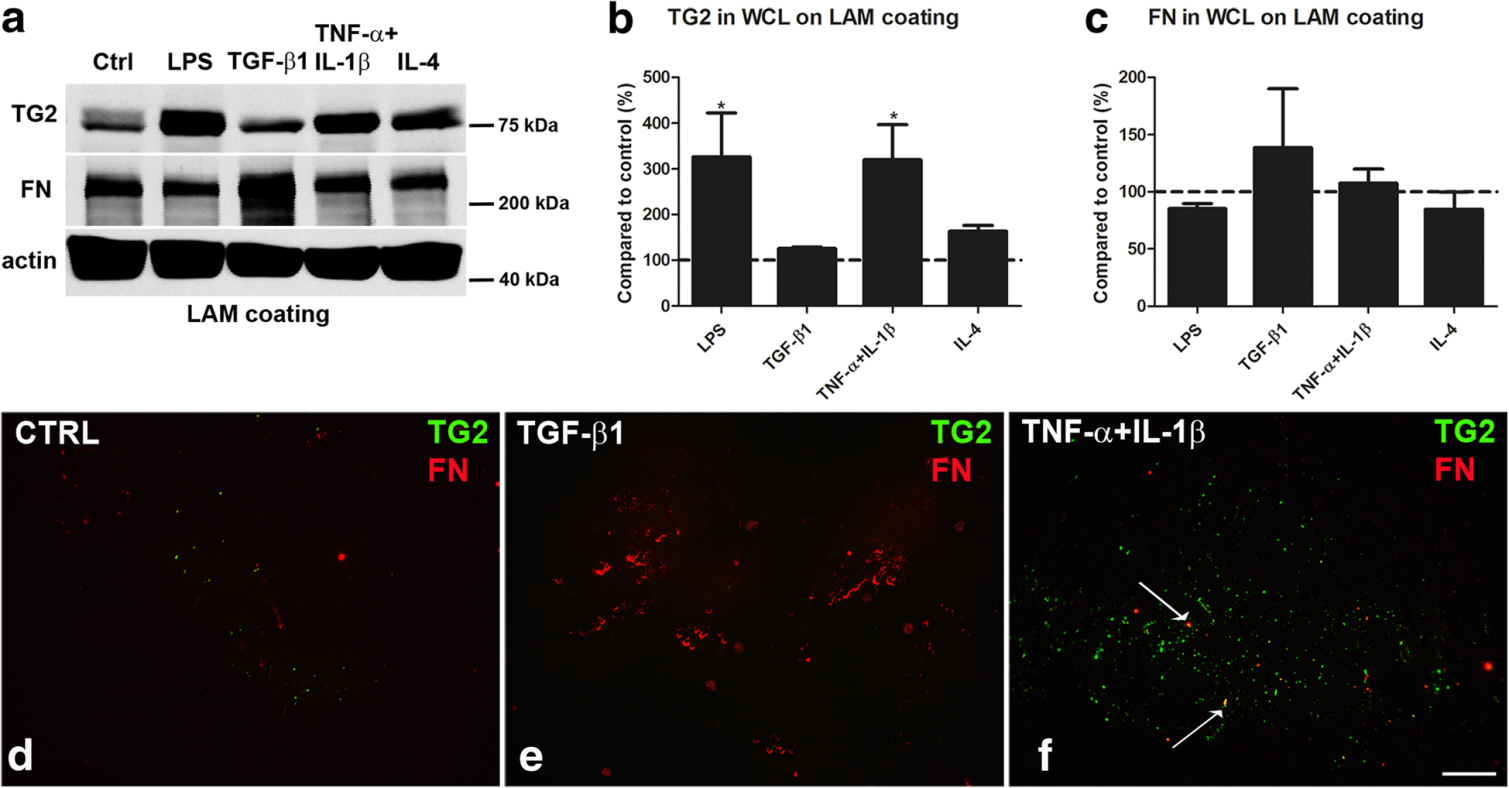
TG2 and fibronectin are deposited in the ECM by astrocytes. Primary rat astrocytes were plated onto laminin (LAM) coated plastic and were treated for 48 h with a range of inflammatory mediators. TG2 and fibronectin (FN) protein levels in whole cell lysates (WCL) of astrocytes were detected by western blotting (**a**), which were subsequently semi-quantified (**b, c**). Each bar represents the mean + standard error of the mean of signal intensities from blots of three separate experiments that were calculated as average percentage compared to control (*P < 0.05). Primary rat astrocytes were treated for 48 h with medium alone as a control condition (CTRL), TGF-β1 or TNF-α+IL-1β, after which astrocytes were removed. TG2 deposition (green) and fibronectin deposition (FN, red) were examined by immunohistochemical staining of the extracellular matrix (**d**, **e**, and **f**). TG2 and fibronectin showed co-localization in the extracellular matrix after TNF-α+IL-1β treatment (indicated by arrows in **f**). Representative images of three independent experiments are shown. Scale bar represents 50 μm

Astrocytes were subsequently plated onto the ECM protein laminin to mimic the situation of an MS lesion, where laminin expression is increased [22]. Moreover, laminin is of interest because it is expressed by cultured astrocytes from healthy subjects and MS patients [29], and laminin is a myelination permissive ECM molecule [68–70]. Under those conditions, we again examined the effect of inflammatory conditions on TG2 and fibronectin levels. LPS or TNF-α+IL-1β treatment were as effective in enhancing TG2 and fibronectin protein levels (*P* < 0.05 for both treatments, Fig. 3a–c) as they were in astrocytes cultured on PLL (Fig. 1). However, IL-4 did not affect TG2 protein levels in astrocytes cultured on laminin (Fig. 3a, b) in contrast to astrocytes cultured on PLL (Fig. 1a, b), while fibronectin levels were not affected in astrocytes cultured on both ECM proteins (Fig. 1a, c and Fig. 3a, c). TGF-β1 treatment did not affect TG2 protein levels in astrocytes cultured on either PLL or laminin (Fig. 1a, b and Fig. 3a, b), whereas fibronectin protein levels in astrocytes cultured on laminin were slightly enhanced (Fig. 3a, c) but more significantly when astrocytes were cultured on PLL (Fig. 1a, c). We next examined the effect of different inflammatory conditions on the extracellular deposition of endogenously produced TG2 and fibronectin by astrocytes cultured on laminin. Astrocytes were therefore first cultured on laminin coated chamber slides and treated with TGF-β1 or a combination of TNF-α+IL-1β cytokines for 48 h. The astrocytes were then removed and the ECM, produced by these astrocytes during the 48 h incubation period, was immunohistochemically stained for TG2 and fibronectin. Without treatment, little extracellular TG2 and fibronectin immunoreactivity in the ECM could be detected (Fig. 3d). Similarly, after TGF-β1 treatment of the cells, TG2 in the ECM was almost undetectable (Fig. 3e), but fibronectin deposition was clearly visible (Fig. 3e) and showed fibril-like structures. Upon treatment with TNF-α+IL-1β, extracellular TG2 immunoreactivity was present in the ECM (Fig. 3f). This treatment did not visibly affect the amount of fibronectin deposited in the ECM, though co-localization between TG2 and fibronectin was apparent under this condition (indicated by arrows in Fig. 3f).

### Exogenous TG2 affected astrocyte-derived fibronectin deposition and cross-linking

**Fig. 4:**
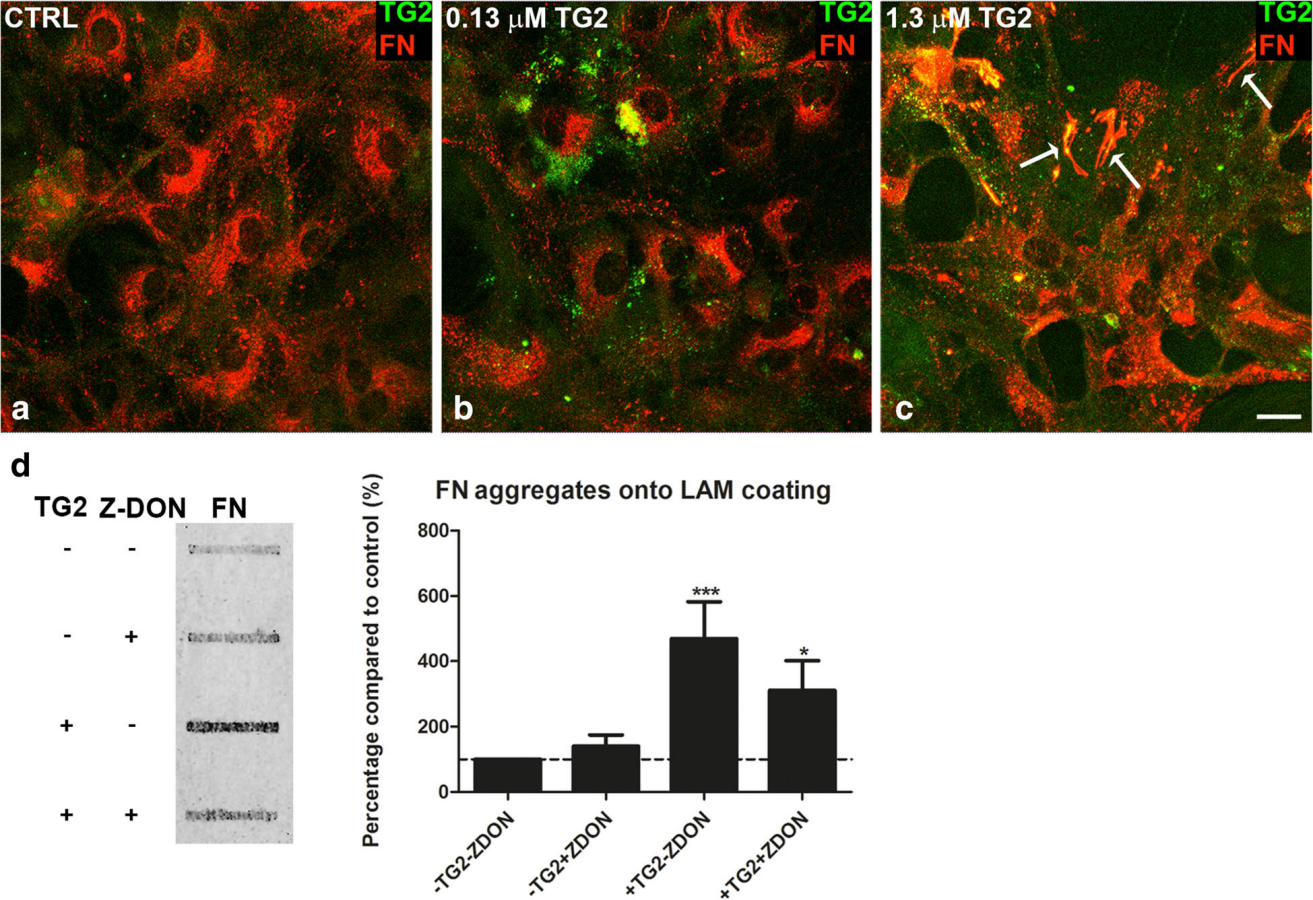
Exogenous TG2 affects astrocyte-derived fibronectin deposition and cross-linking. Primary rat astrocytes were plated on laminin coated plastic and double immunocytochemical surface stainings detected fibronectin (FN, red) and TG2 (green) in untreated control astrocytes (CTRL) and in astrocytes that were treated with exogenous TG2 (0.13 or 1.3 μM) for 48 h (**a**, **b**, and **c**). Exogenous TG2 resulted in morphologically altered fibronectin deposition (indicated by arrows in **c**). Representative images of three independent experiments are shown. Scale bar represents 20 μm. Fibronectin (FN) aggregates on laminin (LAM) coated plastic were studied after removal of untreated astrocytes that were allowed to produce extracellular matrix for 48 h. The extracellular matrix was incubated with exogenous TG2 (0.64 μM, 16 h) and pre-incubated with the specific TG2 inhibitor Z-DON (1 μM) for 30 min at 37 °C (**d**). Representative blots of four independent experiments are shown. Each bar represents the mean + standard error of the mean of signal intensities from blots of four separate experiments that were calculated as average percentage compared to control (*P < 0.05 and ***P < 0.001)

To follow-up on the observation that TG2 can be extracellular deposited by astrocytes, we determined if extracellular TG2 can have an effect on fibronectin deposition. As proof of principle, we applied exogenous TG2 to untreated astrocytes plated onto laminin and studied fibronectin deposition and aggregation. We observed that within a pathophysiological range of TG2 concentrations, the concentration of 0.13 μM exogenously added TG2 was visible in either the ECM or interacting with astrocytes, but no visible effect on fibronectin expression or deposition was observed (Fig. 4b). When the TG2 concentration of 1.3 μM was added to the cells, it resulted in a more general interaction with astrocytes and was possibly incorporated into the astrocytes (Fig. 4c). In addition, this concentration of TG2 resulted in morphologically altered fibronectin deposition which appeared with a more fibrillary structure (as indicated by arrows in Fig. 4c). Moreover, adding 0.64 μM TG2 to the deposited ECM, after removal of astrocytes, resulted in a significant increase in astrocyte-derived fibronectin aggregates in the ECM as detected by FTA (*P* < 0.001, Fig. 4d). Pre-incubation of the ECM with the specific TG2 inhibitor Z-DON partially, but significantly, reduced this increase in fibronectin aggregates (*P* < 0.05, Fig. 4d), indicating that TG2 activity contributes to an altered morphology of the fibronectin deposited and to fibronectin aggregation.

### Endogenous TG2 affected the morphological appearance of fibronectin

**Fig. 5:**
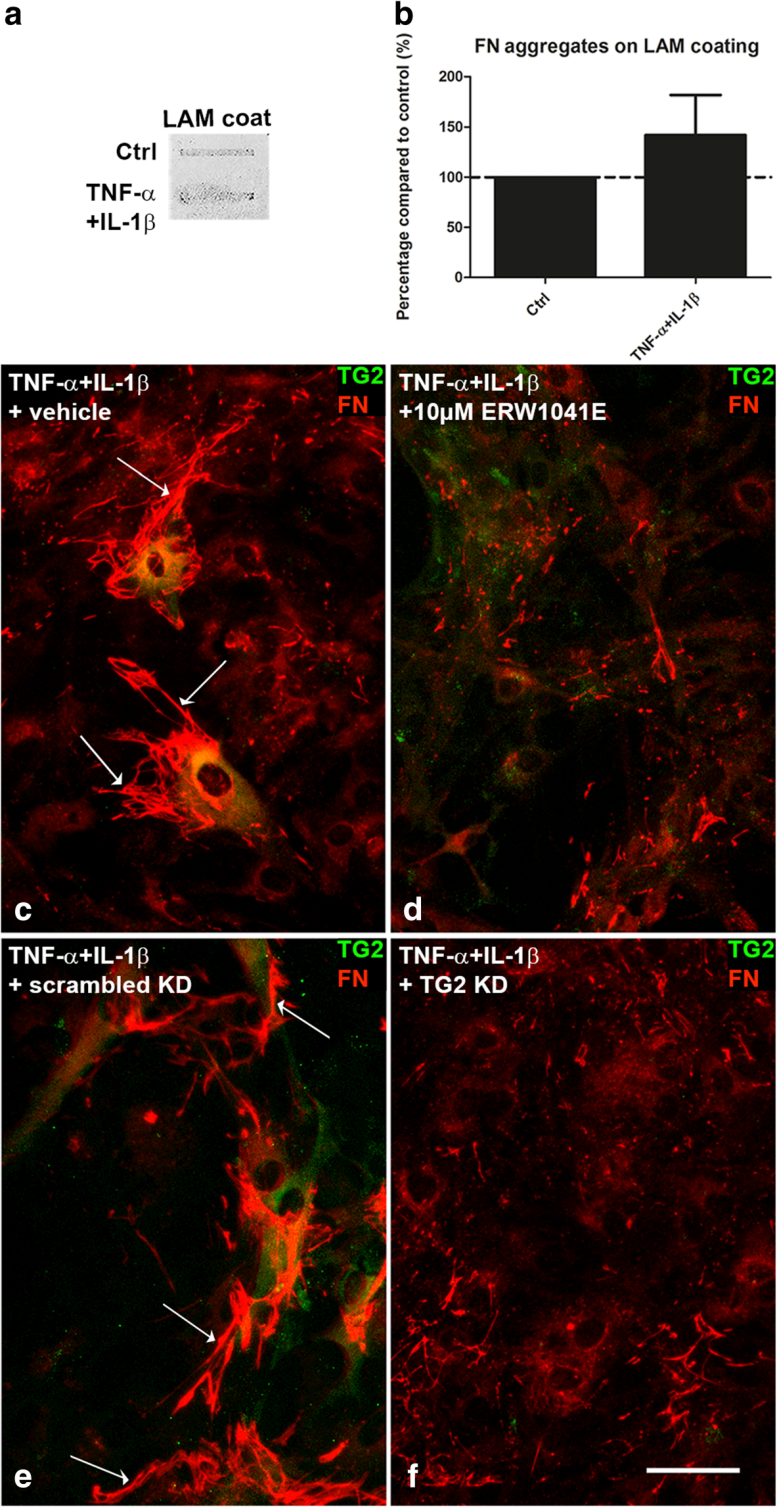
Endogenous TG2 affects the morphological appearance of fibronectin. Fibronectin (FN) aggregates on laminin (LAM) coated plastic were studied after removal of astrocytes that were allowed to produce extracellular matrix during 48 h of TNF-α+IL-1β treatment (**a**, **b**). Representative blots of four independent experiments are shown. Each bar represents the mean + standard error of the mean of signal intensities from blots of four separate experiments that were calculated as average percentage compared to control. Primary rat astrocytes were treated with TNF-α+IL-1β for 48 h plated on laminin coated plastic. Immunocytochemical double stainings showed immunoreactivity of fibronectin (FN, red) and TG2 (green) in astrocytes (**c**, **d**, **e**, and **f**). Astrocytes were co-incubated with TNF-α+IL-1β and the TG2-specific inhibitor ERW1041E (10 μM for 48 h) or DMSO (**d**) or treated with TNF-α+IL-1β after lentiviral downregulation of TG2 (TG2 knockdown (KD)) compared to scrambled knockdown (scrambled KD) (**f**). Fibril-like fibronectin deposition (indicated by arrows in **c** and **e**) was morphological altered after treatment with ERW1041E and after lentiviral downregulation of TG2. Scale bar represents 20 μm

We next determined whether endogenously produced TG2, enhanced by MS relevant inflammatory stimuli, is involved in fibronectin deposition and aggregation. As already found that TNF-α+IL-1β did not affect fibronectin protein levels in astrocytes (Fig. 3c), we now observed that it neither significantly affected aggregation of extracellular fibronectin (Fig. 5a, b). Interestingly, the morphological appearance of extracellular fibril-like fibronectin deposits (Fig. 5c, see arrows) was clearly altered, i.e., shorter and thinner fibrils, upon co-incubation with the bona fide TG2 inhibitor ERW1041E (48 h) (Fig. 5d). Also, after lentiviral downregulation of TG2 (37% TG2 protein left of control scrambled shRNA treatment, data not shown), the morphological fibril-like fibronectin deposition, as found in astrocytes lentivirally transduced with control scrambled shRNA (Fig. 5e, see arrows), was altered (Fig. 5f).

## Discussion

In the present study, we demonstrated that certain inflammatory mediators enhance TG2 protein levels in astrocytes and microglial cells. In addition, fibronectin protein levels were elevated in astrocytes, but not microglial cells. Although exogenous TG2 contributed to aggregation of fibronectin produced by astrocytes, endogenously produced TG2 contributed to the appearance of morphological fibril-like fibronectin, but was not involved in fibronectin aggregation under inflammatory conditions in vitro.

At first, the present study extends our previous observations in astroglia to microglia and additional inflammatory treatment effects were studied. We now observed that astrocytes produced more TG2 than microglia. To determine which inflammatory mediators affect TG2 protein levels in rat astrocytes and microglial cells, the effect of a wide range of inflammatory mediators was studied. Of these LPS, TNF-α+IL-1β and IL-4 most prominently enhanced TG2 protein levels in both cell types, with a higher fold increase in astrocytes. It has been shown before by us and others that astrocytes can produce TG2 [50, 71, 72]. A recent study indicated that mouse microglial cells and not astrocytes are the primary source of TG2 mRNA [73]. Their contrasting observation may be due to differences in species, rats in our study and mice in theirs, and developmental stage of the cells that can affect mRNA expression [74–76]. The responsiveness of glial cells to various inflammatory mediators can be explained by the presence of inflammatory factor-related response elements in the promotor region of the TG2 gene [47, 77, 78], including NF-κB (nuclear factor-kappa B), a transcription factor involved in the regulation of expression of many inflammatory mediators [79].

Astrogliosis is considered to contribute to the scar formation in MS lesions. As part of that process, astro- and microglial cells produce ECM proteins to stabilize the matrix which then becomes a non-permissive environment for regeneration, by depositing ECM proteins that are often inhibitory to regeneration, e.g., chondroitin sulfate proteoglycans [24, 51, 80], hyaluronan [19, 81, 82], and fibronectin [29, 30, 69]. In the present study, we observed that rat astrocytes had higher fibronectin protein levels than microglial cells. This is in line with other observations showing that ECM proteins are predominantly produced by astrocytes and to a lesser extent by microglia and other glial cells [8, 29, 52, 53].

We thus subsequently focused on the regulation of TG2 and fibronectin production in astrocytes during inflammatory conditions. Besides intracellular expression of TG2, we observed TG2 protein on the surface of astrocytes which was also regulated by inflammatory mediators (data not shown). This is in agreement with our previous finding that TG2 is upregulated in the presence of IL-1β and TNF-α at the cell surface of rat astrocytes, where we showed that this coincided with an increased adhesion to and interaction of astrocytes with the ECM protein fibronectin, creating an environment for astroglial scarring [50]. In addition to TG2 protein production, its activity is essential to mediate possible cross-linking of fibronectin. We now showed that TG2 activity was present and increased by LPS or TNF-α+IL-1β, but was not significantly affected by IL-4 treatment. This last observation was unexpected, as the amount of TG2 protein was enhanced, and in human macrophages, IL-4 does increase TG2 activity [83]. This may suggest that in astrocytes, IL-4-generated TG2 has a function different from protein cross-linking. In agreement with our finding that astrocytes respond to inflammatory conditions by increasing their TG2 expression and activity, others have shown, in different cell types and tissues, such as lung and liver cells and cartilage tissue, that inflammatory cytokines, including TNF-α and IL-1β, can indeed upregulate TG2 expression and activity [47, 77, 84–86].

It is of interest to note that the effects some cytokines had on astrocytes that were cultured on PLL, such as an increase in TG2 protein levels after IL-4 treatment and an increase in fibronectin protein levels after TGF-β1 treatment, were no longer present when astrocytes were cultured on laminin. This is in line with previous work that showed that ECM composition determines astrocyte responses to inflammatory stimuli [87] and extends on our previous observations [50]. As in brain lesions induced by damage, i.e., MS, more ECM proteins such as laminin are present [22, 27], we considered it relevant to pursue our studies with astrocytes exposed to laminin in the culture dish. The subsequent effect of inflammatory mediators in our study on either TG2 or fibronectin was not confined to protein expression inside the cells, but extracellular deposition of both TG2 and fibronectin was also affected by TNF-α+IL-1β and TGF-β1. Under inflammatory conditions, including MS, it is well known that astrocytes contribute to ECM production and deposition [8, 13, 21, 88–90]. In our present study, we showed that the addition of exogenous TG2 to astrocytes resulted in a fibril-like morphology of the fibronectin deposited and increased fibronectin aggregation. This protein aggregation was partially reduced by the TG2-specific inhibitor Z-DON, which suggests that TG2 activity can, at least partly, mediate aggregation of astrocyte-derived fibronectin, shown here as fibronectin fibrils. Alternatively, a non-enzymatic, non-covalent interaction of TG2 with fibronectin [91] might contribute to the altered morphology of the fibronectin. Such TG2-fibronectin interaction stabilizes the ECM by enhancing fibronectin matrix formation and interaction with other proteins, such as integrins, syndecan-4, growth factor receptors, and other cell surface or ECM proteins [32, 92–94].

Under inflammatory conditions, as present in MS lesions, when endogenous TG2 protein levels were enhanced by TNF-α+IL-1β treatment of astrocytes, fibronectin deposits showed morphologically a fibril-like structure. This was similar as shown by addition of exogenous TG2, while now fibronectin aggregation was absent. These observations are suggestive for a role of TG2 in the assembly of fibronectin into a fibril-like structure without being aggregated. Indeed, treatment of astrocytes with the TG2 inhibitor ERW1041E or lentiviral downregulation of TG2 with TG2-specific shRNA in astrocytes altered the morphology of TNF-α+IL-1β-induced fibronectin deposits, by reducing fibril size and thickness, indicating that TG2 is involved in this process in vitro. Though, no clear fibronectin aggregates were observed, which were evident when TG2 was exogenously added to the astrocytes. This is in line with the observations that TG2 can lead to a switch from intramolecular cross-linking to intermolecular protein cross-linking giving rise to protein aggregate formation [63]. Thus, we cannot exclude that the level of TG2 protein present in astrocytes under inflammatory conditions in vitro is able to alter the conformation of fibronectin to a fibril-like structure, but it is not able to form cross-linked protein aggregates. In support of this, we also did not observe fibronectin aggregation in the cuprizone model of demyelination in which astrocytes become hypertrophic and express TG2 and fibronectin [49]. However, fibronectin deposition is found in MS lesions as a complex network of fibrils of high molecular weight aggregates, which impairs remyelination [29]. Fibronectin aggregation, as defined by deoxycholate-insolubility, is likely the result of strong, non-covalent protein-protein interactions [31, 95, 96]. As a consequence, we might hypothesize that during inflammatory lesion formation in MS and concomitant deposition of ECM proteins, astrocyte-derived TG2, only in the presence of infiltrating leukocytes or their derived factors, can increase the formation of fibronectin fibril-like deposits which aggregate and may contribute to the process of scarring and subsequent impaired remyelination.

## Conclusions

The results presented here demonstrate that under inflammatory conditions in vitro, TG2 protein levels are enhanced in astrocytes and microglia. In addition, in particular, astrocytes produce ECM proteins, i.e., fibronectin, that can be cross-linked and aggregated by exogenously added TG2. Endogenously produced TG2, enhanced by inflammatory stimuli, is involved in the appearance of morphological fibril-like fibronectin deposits, but does not lead to cross-linked fibronectin aggregates. Thus, our in vitro observations suggest that during MS lesion formation, when inflammatory mediators are produced, astrocyte-derived TG2 may contribute to ECM rearrangement and subsequent astroglial scarring. The possibility to intervene with the formation of fibronectin fibril-like deposits may open avenues to reduce the astroglial scarring process, not only in MS but also in other CNS disorders, when scarring prohibits regeneration [97].

## Additional file

Additional file 1: TG2 and fibronectin protein expression in rat astrocytes and microglia. Untreated (Ctrl) and 48 h LPS-treated primary rat astrocytes (A) expressed more fibronectin (FN) and more TG2 compared to untreated (Ctrl) and LPS-treated primary rat microglia (M). Representative blot of three independent experiments is shown. (TIFF 195 kb)

## Abbreviations

biotin-Cd: Biotinylated-cadaverine
CNS: Central nervous system
CTRL: Control
DTT: Dithiothreitol
ECM: Extracellular matrix
FN: Fibronectin
FTA: Filter trap assay
GFAP: Glial fibrillary acidic protein
h: Hours
HRP: Horseradish peroxidase
IL-10: Interleukin-10
IL-1β: Interleukin-1 beta
IL-4: Interleukin-4
IL-6: Interleukin-6
LAM: Laminin
LPS: Lipopolysaccharide
Min: Minutes
MS: Multiple sclerosis
NF-κB: Nuclear factor-kappa B
PLL: Poly-L-lysine
PMSF: Phenylmethanesulfonyl fluoride
RT: Room temperature
SDS: Sodium dodecyl sulfate
TBS: Tris-buffered saline
TBS-T: Tris-buffered saline 0.1%/0.05% Triton
TG: Transglutaminase
TG2: Tissue transglutaminase
TGF-β1: Transforming growth factor-beta 1
TGF-β2: Transforming growth factor-beta 2
TNF-α: Tumor necrosis factor-alpha
