# Supplementary figures and images for: Tissue transglutaminase in astrocytes is enhanced by inflammatory mediators and is involved in the formation of fibronectin fibril-like structures
Source: J Neuroinflammation. 2017 Dec 28;14:260. doi: 10.1186/s12974-017-1031-2 (PMC5745633; doi:10.1186/s12974-017-1031-2)

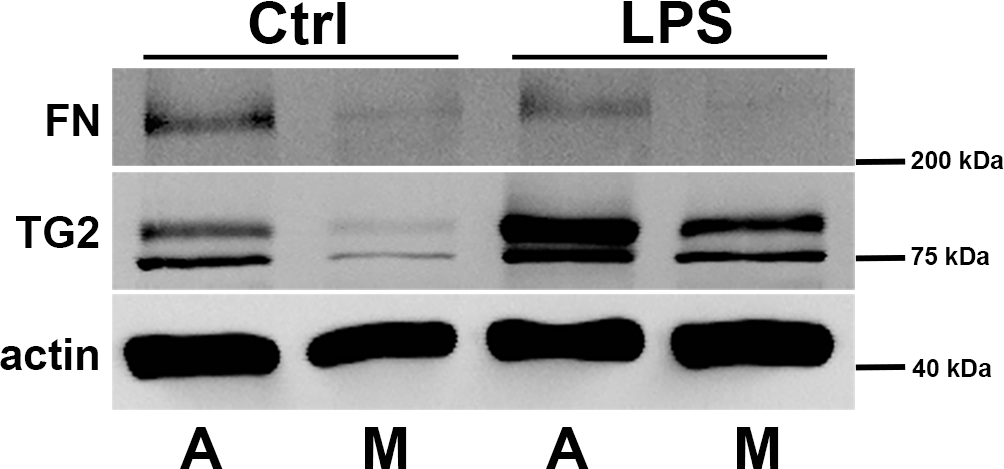

Supplement: Additional file 1: — TG2 and fibronectin protein expression in rat astrocytes and microglia. Untreated (Ctrl) and 48 h LPS-treated primary rat astrocytes (A) expressed more fibronectin (FN) and more TG2 compared to untreated (Ctrl) and LPS-treated primary rat microglia (M). Representative blot of three independent experiments is shown. (TIFF 195 kb) [file 12974_2017_1031_MOESM1_ESM.tif]
